# A New Attack on the Cancer Problem

**Published:** 1909-05-01

**Authors:** Dan McKenzie


### CLINICAL NOTES.

### A NEW ATTACK ON THE CANCER PROBLEM.

By DAN McKENZIE, M.D., F.R.C.S.E.

In an interesting series of articles by Mr. H. C. Ross and Dr. C. J. Macalister * of Liverpool, the attention of those interested in cancer research is diverted from the local disease-process and directed to certain changes in the blood, which these observers suppose, may be responsible for the local outbreak.

Ross, seeking for a method by which living could be differentiated from dead leucocytes, hit upon the plan of supplying excitants to the cells, so that those which are alive give evidence of their vitality by pushing out pseudopodia, while those which are dead lie inert. His fellow-worker thereupon suggested that the blood of cancer patients should be examined, by bringing it into contact with healthy leucocytes, in order to ascertain whether or not it contains a similar excitant of leucocytic activity. Curiously enough, it was found that the blood-plasma of cancerous patients does possess this property. Finally, the discovery was made that the response of lymphocytes to the excitant action of the blood-plasma of cancerous individuals differs from that of leucocytes. In the case of the latter, the irritation induces the protrusion of the familiar pseudopodia; but in the case of the former, the processes of cytoplasm protruded by the lymphocyte have a flagellate appearance, and, bearing as they do, a well-defined granule at their terminal extremity, present a close resemblance to spermatozoa, a resemblance which is heightened when the flagellæ break off from the cell and appear to move about in the surrounding plasma.

Struck by this similarity in appearance to spermatozoa, Ross and Macalister suggest, in a bold flight of the imagination, that these flagellæ may actually possess a fertilising power, in virtue of which, when brought into contact with certain tissue-cells, they may set in motion that form of vegetative reproduction we know by the name of "cancer."

As to the nature of the excitant in the blood which causes the lymphocytes to manifest these phenomena we are in the dark. But the authors remind us that methylene blue and other coal-tar products possess the property of stimulating leucocytic activity, and that exposure to soot, coal-tar, etc., predisposes an individual to cancer. The frequency with which cancer develops in regions of the body exposed to irritation, and in organs which manifest phases of great physiological activity is explained by ascribing it to the presence in the parts so affected of a copious quantity of "reproductive blood"; that is, blood which has the power of stimulating or originating the reproduction of tissue-cells.

* *Lancet*, January 16, 1909, p. 152 and p. 154. *Brit. Med. Journ.*, January 23, 1909, p. 206.

Most of this argument is, of course, pure hypothesis, and, indeed, hypothesis of a very fragile structure in certain respects. Why, for example, should we not look upon the activity of leucocytes and lymphocytes in the presence of cancerous blood-plasma as evidence rather of a protective effort on the part of the blood-scavengers, than of a method by which the cancerous growth may be induced? It is generally recognised that cancer-cells which find their way into the blood quickly perish.

At the same time the argument is so bold and suggestive that it deserves, and doubtless will receive, a better fate than to be annihilated by theoretical objections. In the difficult problem of cancer, as in every other investigation of nature, it is the imagination that opens up to us new avenues of approach. We may, therefore, permit ourselves to hope, with some amount of confidence, that Messrs. Ross and Macalister, not only by their observations and discoveries, but also by their stimulating theories, have initiated an attack, pushed forward from a fresh base, which may bring us nearer to the removal of the reproach of scientific medicine—cancer.

